# Supplementary material for: Dynalign II: common secondary structure prediction for RNA homologs with domain insertions
Source: Nucleic Acids Res. 2014 Nov 21;42(22):13939–48. doi: 10.1093/nar/gku1172 (PMC4267632; doi:10.1093/nar/gku1172)
Supplement: SUPPLEMENTARY DATA [file supp_gku1172_nar-02021-z-2014-File012.zip › manual/javadoc/constant-values.html]

Constant Field Values


JavaScript is disabled on your browser.


- Overview
- Package
- Class
- Tree
- Index
- Help

- Prev
- Next

- Frames
- No Frames

- All Classes

# Constant Field Values

## Contents

- RNAstructure\_java\_drawing.source.\*
- RNAstructure\_java\_interface.source.\*
- RNAstructure\_java\_utilities.\*

## RNAstructure\_java\_drawing.source.\*

- RNAstructure\_java\_drawing.source.dialogs.ImageDialog

  | Modifier and Type | Constant Field | Value |
  | --- | --- | --- |
  |  |  |  |
  | --- | --- | --- |
  | `protected final int` | `preferredDialogWidth` | `450` |
  | `private static final long` | `serialVersionUID` | `20120802L` |
- RNAstructure\_java\_drawing.source.dialogs.RNAstructure\_java\_drawing.source.dialogs.ImageDialog.PrintMenu

  | Modifier and Type | Constant Field | Value |
  | --- | --- | --- |
  |  |  |  |
  | --- | --- | --- |
  | `private static final long` | `serialVersionUID` | `20120802L` |
- RNAstructure\_java\_drawing.source.dialogs.RNAstructure\_java\_drawing.source.dialogs.ImageDialog.ZoomDialog

  | Modifier and Type | Constant Field | Value |
  | --- | --- | --- |
  |  |  |  |
  | --- | --- | --- |
  | `private static final long` | `serialVersionUID` | `20120802L` |
- RNAstructure\_java\_drawing.source.dialogs.PlotDialog

  | Modifier and Type | Constant Field | Value |
  | --- | --- | --- |
  |  |  |  |
  | --- | --- | --- |
  | `private static final long` | `serialVersionUID` | `20120802L` |
- RNAstructure\_java\_drawing.source.dialogs.StructureDialog

  | Modifier and Type | Constant Field | Value |
  | --- | --- | --- |
  |  |  |  |
  | --- | --- | --- |
  | `private static final long` | `serialVersionUID` | `20120802L` |

- RNAstructure\_java\_drawing.source.menus.AnnotationMenu

  | Modifier and Type | Constant Field | Value |
  | --- | --- | --- |
  |  |  |  |
  | --- | --- | --- |
  | `private static final long` | `serialVersionUID` | `20120802L` |
- RNAstructure\_java\_drawing.source.menus.DotsMenu

  | Modifier and Type | Constant Field | Value |
  | --- | --- | --- |
  |  |  |  |
  | --- | --- | --- |
  | `private static final long` | `serialVersionUID` | `20120615L` |
- RNAstructure\_java\_drawing.source.menus.RNAstructure\_java\_drawing.source.menus.DotsMenu.ColorDialog

  | Modifier and Type | Constant Field | Value |
  | --- | --- | --- |
  |  |  |  |
  | --- | --- | --- |
  | `private static final long` | `serialVersionUID` | `20120802L` |
- RNAstructure\_java\_drawing.source.menus.RNAstructure\_java\_drawing.source.menus.DotsMenu.RangeDialog

  | Modifier and Type | Constant Field | Value |
  | --- | --- | --- |
  |  |  |  |
  | --- | --- | --- |
  | `private static final long` | `serialVersionUID` | `20120802L` |
- RNAstructure\_java\_drawing.source.menus.PlotMenu

  | Modifier and Type | Constant Field | Value |
  | --- | --- | --- |
  |  |  |  |
  | --- | --- | --- |
  | `private static final long` | `serialVersionUID` | `20120615L` |
- RNAstructure\_java\_drawing.source.menus.StructureMenu

  | Modifier and Type | Constant Field | Value |
  | --- | --- | --- |
  |  |  |  |
  | --- | --- | --- |
  | `private static final long` | `serialVersionUID` | `20120802L` |
- RNAstructure\_java\_drawing.source.menus.RNAstructure\_java\_drawing.source.menus.StructureMenu.MoveDialog

  | Modifier and Type | Constant Field | Value |
  | --- | --- | --- |
  |  |  |  |
  | --- | --- | --- |
  | `private static final long` | `serialVersionUID` | `20120802L` |

- RNAstructure\_java\_drawing.source.runners.PlotRunner

  | Modifier and Type | Constant Field | Value |
  | --- | --- | --- |
  |  |  |  |
  | --- | --- | --- |
  | `private static final long` | `serialVersionUID` | `20120802L` |
- RNAstructure\_java\_drawing.source.runners.StructureRunner

  | Modifier and Type | Constant Field | Value |
  | --- | --- | --- |
  |  |  |  |
  | --- | --- | --- |
  | `private static final long` | `serialVersionUID` | `20120802L` |


## RNAstructure\_java\_interface.source.\*

- RNAstructure\_java\_interface.source.menus.ConstraintsMenu

  | Modifier and Type | Constant Field | Value |
  | --- | --- | --- |
  |  |  |  |
  | --- | --- | --- |
  | `private static final long` | `serialVersionUID` | `20120802L` |
- RNAstructure\_java\_interface.source.menus.ConstraintsMenu.NucOrHelixDialog

  | Modifier and Type | Constant Field | Value |
  | --- | --- | --- |
  |  |  |  |
  | --- | --- | --- |
  | `private static final long` | `serialVersionUID` | `20120802L` |
- RNAstructure\_java\_interface.source.menus.DynamicMenuBar

  | Modifier and Type | Constant Field | Value |
  | --- | --- | --- |
  |  |  |  |
  | --- | --- | --- |
  | `private static final long` | `serialVersionUID` | `20120802L` |
- RNAstructure\_java\_interface.source.menus.FileMenu

  | Modifier and Type | Constant Field | Value |
  | --- | --- | --- |
  |  |  |  |
  | --- | --- | --- |
  | `private static final long` | `serialVersionUID` | `20120802L` |
- RNAstructure\_java\_interface.source.menus.HelpMenu

  | Modifier and Type | Constant Field | Value |
  | --- | --- | --- |
  |  |  |  |
  | --- | --- | --- |
  | `private static final long` | `serialVersionUID` | `20120802L` |
- RNAstructure\_java\_interface.source.menus.NucleicAcidMenu

  | Modifier and Type | Constant Field | Value |
  | --- | --- | --- |
  |  |  |  |
  | --- | --- | --- |
  | `private static final long` | `serialVersionUID` | `20120802L` |
- RNAstructure\_java\_interface.source.menus.RolloverMenu

  | Modifier and Type | Constant Field | Value |
  | --- | --- | --- |
  |  |  |  |
  | --- | --- | --- |
  | `private static final long` | `serialVersionUID` | `20120802L` |
- RNAstructure\_java\_interface.source.menus.RolloverMenu.RolloverListener

  | Modifier and Type | Constant Field | Value |
  | --- | --- | --- |
  |  |  |  |
  | --- | --- | --- |
  | `private static final long` | `serialVersionUID` | `20120802L` |

- RNAstructure\_java\_interface.source.startup.ApplicationRootFrame

  | Modifier and Type | Constant Field | Value |
  | --- | --- | --- |
  |  |  |  |
  | --- | --- | --- |
  | `private static final long` | `serialVersionUID` | `20120802L` |
- RNAstructure\_java\_interface.source.startup.RNAstructure

  | Modifier and Type | Constant Field | Value |
  | --- | --- | --- |
  |  |  |  |
  | --- | --- | --- |
  | `private static final long` | `serialVersionUID` | `20120802L` |

- RNAstructure\_java\_interface.source.windows.DrawingWindow

  | Modifier and Type | Constant Field | Value |
  | --- | --- | --- |
  |  |  |  |
  | --- | --- | --- |
  | `private static final long` | `serialVersionUID` | `20120802L` |
- RNAstructure\_java\_interface.source.windows.DynalignFoldWindow

  | Modifier and Type | Constant Field | Value |
  | --- | --- | --- |
  |  |  |  |
  | --- | --- | --- |
  | `private static final long` | `serialVersionUID` | `20120802L` |
- RNAstructure\_java\_interface.source.windows.DynalignRefoldWindow

  | Modifier and Type | Constant Field | Value |
  | --- | --- | --- |
  |  |  |  |
  | --- | --- | --- |
  | `private static final long` | `serialVersionUID` | `20120802L` |
- RNAstructure\_java\_interface.source.windows.Efn2Window

  | Modifier and Type | Constant Field | Value |
  | --- | --- | --- |
  |  |  |  |
  | --- | --- | --- |
  | `private static final long` | `serialVersionUID` | `20120802L` |
- RNAstructure\_java\_interface.source.windows.FoldDoubleWindow

  | Modifier and Type | Constant Field | Value |
  | --- | --- | --- |
  |  |  |  |
  | --- | --- | --- |
  | `private static final long` | `serialVersionUID` | `20120802L` |
- RNAstructure\_java\_interface.source.windows.FoldSingleWindow

  | Modifier and Type | Constant Field | Value |
  | --- | --- | --- |
  |  |  |  |
  | --- | --- | --- |
  | `private static final long` | `serialVersionUID` | `20120802L` |
- RNAstructure\_java\_interface.source.windows.FoldSuboptimalWindow

  | Modifier and Type | Constant Field | Value |
  | --- | --- | --- |
  |  |  |  |
  | --- | --- | --- |
  | `private static final long` | `serialVersionUID` | `20120802L` |
- RNAstructure\_java\_interface.source.windows.InternalWindow

  | Modifier and Type | Constant Field | Value |
  | --- | --- | --- |
  |  |  |  |
  | --- | --- | --- |
  | `private static final long` | `serialVersionUID` | `20120802L` |
- RNAstructure\_java\_interface.source.windows.MaxExpectWindow

  | Modifier and Type | Constant Field | Value |
  | --- | --- | --- |
  |  |  |  |
  | --- | --- | --- |
  | `private static final long` | `serialVersionUID` | `20120802L` |
- RNAstructure\_java\_interface.source.windows.ModuleWindow

  | Modifier and Type | Constant Field | Value |
  | --- | --- | --- |
  |  |  |  |
  | --- | --- | --- |
  | `private static final long` | `serialVersionUID` | `20120802L` |
- RNAstructure\_java\_interface.source.windows.MultilignWindow

  | Modifier and Type | Constant Field | Value |
  | --- | --- | --- |
  |  |  |  |
  | --- | --- | --- |
  | `private static final long` | `serialVersionUID` | `20120802L` |
- RNAstructure\_java\_interface.source.windows.MultiWindow

  | Modifier and Type | Constant Field | Value |
  | --- | --- | --- |
  |  |  |  |
  | --- | --- | --- |
  | `protected final int` | `columnWidth` | `350` |
  | `private static final long` | `serialVersionUID` | `20120802L` |
- RNAstructure\_java\_interface.source.windows.OligoScreenWindow

  | Modifier and Type | Constant Field | Value |
  | --- | --- | --- |
  |  |  |  |
  | --- | --- | --- |
  | `private static final long` | `serialVersionUID` | `20120802L` |
- RNAstructure\_java\_interface.source.windows.OligoWalkWindow

  | Modifier and Type | Constant Field | Value |
  | --- | --- | --- |
  |  |  |  |
  | --- | --- | --- |
  | `private static final long` | `serialVersionUID` | `20120802L` |
- RNAstructure\_java\_interface.source.windows.RNAstructure\_java\_interface.source.windows.OligoWalkWindow.BarButton

  | Modifier and Type | Constant Field | Value |
  | --- | --- | --- |
  |  |  |  |
  | --- | --- | --- |
  | `private static final long` | `serialVersionUID` | `20120802L` |
- RNAstructure\_java\_interface.source.windows.OligoWalkWindow.OligoSelectionDialog

  | Modifier and Type | Constant Field | Value |
  | --- | --- | --- |
  |  |  |  |
  | --- | --- | --- |
  | `private static final long` | `serialVersionUID` | `20120802L` |
- RNAstructure\_java\_interface.source.windows.RNAstructure\_java\_interface.source.windows.OligoWalkWindow.OligoWalkResultsMenu

  | Modifier and Type | Constant Field | Value |
  | --- | --- | --- |
  |  |  |  |
  | --- | --- | --- |
  | `private static final long` | `serialVersionUID` | `20120802L` |
- RNAstructure\_java\_interface.source.windows.OligoWalkWindow.SpinnerPanel

  | Modifier and Type | Constant Field | Value |
  | --- | --- | --- |
  |  |  |  |
  | --- | --- | --- |
  | `private static final long` | `serialVersionUID` | `20120802L` |
- RNAstructure\_java\_interface.source.windows.OligoWalkWindow.StringComboBox

  | Modifier and Type | Constant Field | Value |
  | --- | --- | --- |
  |  |  |  |
  | --- | --- | --- |
  | `private static final long` | `serialVersionUID` | `20120802L` |
- RNAstructure\_java\_interface.source.windows.PartitionDoubleWindow

  | Modifier and Type | Constant Field | Value |
  | --- | --- | --- |
  |  |  |  |
  | --- | --- | --- |
  | `private static final long` | `serialVersionUID` | `20120802L` |
- RNAstructure\_java\_interface.source.windows.PartitionSingleWindow

  | Modifier and Type | Constant Field | Value |
  | --- | --- | --- |
  |  |  |  |
  | --- | --- | --- |
  | `private static final long` | `serialVersionUID` | `20120802L` |
- RNAstructure\_java\_interface.source.windows.ProbKnotWindow

  | Modifier and Type | Constant Field | Value |
  | --- | --- | --- |
  |  |  |  |
  | --- | --- | --- |
  | `private static final long` | `serialVersionUID` | `20120802L` |
- RNAstructure\_java\_interface.source.windows.PseudoknotWindow

  | Modifier and Type | Constant Field | Value |
  | --- | --- | --- |
  |  |  |  |
  | --- | --- | --- |
  | `private static final long` | `serialVersionUID` | `20120802L` |
- RNAstructure\_java\_interface.source.windows.RefoldWindow

  | Modifier and Type | Constant Field | Value |
  | --- | --- | --- |
  |  |  |  |
  | --- | --- | --- |
  | `private static final long` | `serialVersionUID` | `20120802L` |
- RNAstructure\_java\_interface.source.windows.SequenceDisplayWindow

  | Modifier and Type | Constant Field | Value |
  | --- | --- | --- |
  |  |  |  |
  | --- | --- | --- |
  | `private static final long` | `serialVersionUID` | `20120802L` |
- RNAstructure\_java\_interface.source.windows.RNAstructure\_java\_interface.source.windows.SequenceDisplayWindow.EditMenu

  | Modifier and Type | Constant Field | Value |
  | --- | --- | --- |
  |  |  |  |
  | --- | --- | --- |
  | `private static final long` | `serialVersionUID` | `20120802L` |
- RNAstructure\_java\_interface.source.windows.RNAstructure\_java\_interface.source.windows.SequenceDisplayWindow.ReadMenu

  | Modifier and Type | Constant Field | Value |
  | --- | --- | --- |
  |  |  |  |
  | --- | --- | --- |
  | `private static final long` | `serialVersionUID` | `20120802L` |
- RNAstructure\_java\_interface.source.windows.StochasticWindow

  | Modifier and Type | Constant Field | Value |
  | --- | --- | --- |
  |  |  |  |
  | --- | --- | --- |
  | `private static final long` | `serialVersionUID` | `20120802L` |
- RNAstructure\_java\_interface.source.windows.TurboFoldWindow

  | Modifier and Type | Constant Field | Value |
  | --- | --- | --- |
  |  |  |  |
  | --- | --- | --- |
  | `private static final long` | `serialVersionUID` | `20120802L` |


## RNAstructure\_java\_utilities.\*

- RNAstructure\_java\_utilities.BorderBuilder

  | Modifier and Type | Constant Field | Value |
  | --- | --- | --- |
  |  |  |  |
  | --- | --- | --- |
  | `private static final long` | `serialVersionUID` | `20120802L` |
- RNAstructure\_java\_utilities.BorderedButtonPanel

  | Modifier and Type | Constant Field | Value |
  | --- | --- | --- |
  |  |  |  |
  | --- | --- | --- |
  | `private static final long` | `serialVersionUID` | `20120802L` |
- RNAstructure\_java\_utilities.FieldPanel

  | Modifier and Type | Constant Field | Value |
  | --- | --- | --- |
  |  |  |  |
  | --- | --- | --- |
  | `private static final long` | `serialVersionUID` | `20120802L` |
- RNAstructure\_java\_utilities.FieldPanel.FilePanel

  | Modifier and Type | Constant Field | Value |
  | --- | --- | --- |
  |  |  |  |
  | --- | --- | --- |
  | `private static final long` | `serialVersionUID` | `20120802L` |
- RNAstructure\_java\_utilities.FieldPanel.NumberPanel

  | Modifier and Type | Constant Field | Value |
  | --- | --- | --- |
  |  |  |  |
  | --- | --- | --- |
  | `private static final long` | `serialVersionUID` | `20120802L` |
- RNAstructure\_java\_utilities.FileChooser

  | Modifier and Type | Constant Field | Value |
  | --- | --- | --- |
  |  |  |  |
  | --- | --- | --- |
  | `private static final long` | `serialVersionUID` | `20120802L` |
- RNAstructure\_java\_utilities.FileField

  | Modifier and Type | Constant Field | Value |
  | --- | --- | --- |
  |  |  |  |
  | --- | --- | --- |
  | `private static final long` | `serialVersionUID` | `20120802L` |
- RNAstructure\_java\_utilities.HTMLCheckBox

  | Modifier and Type | Constant Field | Value |
  | --- | --- | --- |
  |  |  |  |
  | --- | --- | --- |
  | `private static final long` | `serialVersionUID` | `20120802L` |
- RNAstructure\_java\_utilities.ImageGrabber

  | Modifier and Type | Constant Field | Value |
  | --- | --- | --- |
  |  |  |  |
  | --- | --- | --- |
  | `private static final long` | `serialVersionUID` | `20120802L` |
- RNAstructure\_java\_utilities.MacChecker

  | Modifier and Type | Constant Field | Value |
  | --- | --- | --- |
  |  |  |  |
  | --- | --- | --- |
  | `private static final long` | `serialVersionUID` | `20120802L` |
- RNAstructure\_java\_utilities.NumberField

  | Modifier and Type | Constant Field | Value |
  | --- | --- | --- |
  |  |  |  |
  | --- | --- | --- |
  | `private static final long` | `serialVersionUID` | `20120802L` |
- RNAstructure\_java\_utilities.NumberField.DoubleField

  | Modifier and Type | Constant Field | Value |
  | --- | --- | --- |
  |  |  |  |
  | --- | --- | --- |
  | `private static final long` | `serialVersionUID` | `20120802L` |
- RNAstructure\_java\_utilities.NumberField.FloatField

  | Modifier and Type | Constant Field | Value |
  | --- | --- | --- |
  |  |  |  |
  | --- | --- | --- |
  | `private static final long` | `serialVersionUID` | `20120802L` |
- RNAstructure\_java\_utilities.NumberField.IntegerField

  | Modifier and Type | Constant Field | Value |
  | --- | --- | --- |
  |  |  |  |
  | --- | --- | --- |
  | `private static final long` | `serialVersionUID` | `20120802L` |
- RNAstructure\_java\_utilities.RadioButtonPanel

  | Modifier and Type | Constant Field | Value |
  | --- | --- | --- |
  |  |  |  |
  | --- | --- | --- |
  | `private static final long` | `serialVersionUID` | `20120802L` |
- RNAstructure\_java\_utilities.RNAstructureMenu

  | Modifier and Type | Constant Field | Value |
  | --- | --- | --- |
  |  |  |  |
  | --- | --- | --- |
  | `private static final long` | `serialVersionUID` | `20120802L` |
- RNAstructure\_java\_utilities.ScrollerPane

  | Modifier and Type | Constant Field | Value |
  | --- | --- | --- |
  |  |  |  |
  | --- | --- | --- |
  | `public static final int` | `ALWAYS` | `1` |
  | `public static final int` | `NEVER` | `2` |
  | `private static final long` | `serialVersionUID` | `20120802L` |
- RNAstructure\_java\_utilities.SimpleDialogHandler

  | Modifier and Type | Constant Field | Value |
  | --- | --- | --- |
  |  |  |  |
  | --- | --- | --- |
  | `private static final long` | `serialVersionUID` | `20120802L` |
- RNAstructure\_java\_utilities.ValueSelectionDialog

  | Modifier and Type | Constant Field | Value |
  | --- | --- | --- |
  |  |  |  |
  | --- | --- | --- |
  | `private static final long` | `serialVersionUID` | `20120802L` |

- Overview
- Package
- Class
- Tree
- Index
- Help

- Prev
- Next

- Frames
- No Frames

- All Classes
